# Triphenylphosphine-Passivated Aluminum Nanoparticles: Synthesis and Reactivity in Al/CuO Nanothermites

**DOI:** 10.1021/acsomega.6c05552

**Published:** 2026-09-20

**Authors:** Ming-Hsun Wu, Chao-Wei Huang, Ying-Shou Cheng, Xing-Rong Lu, You-Ci Jian

**Affiliations:** † Department of Mechanical Engineering, National Cheng Kung University, Tainan 70101, Taiwan; ‡ Department of Engineering Science, National Cheng Kung University, Tainan 70101, Taiwan; § Department of Chemical and Materials Engineering, 517768 National Kaohsiung University of Science and Technology, Kaohsiung 80778, Taiwan

## Abstract

This work reports the synthesis of triphenylphosphine (PPh_3_)-passivated aluminum nanoparticles (Al@PPh_3_) via a one-pot wet chemical reduction of AlCl_3_ with LiAlH_4_ in refluxing mesitylene at a 1:1 PPh_3_/AlCl_3_ molar ratio under nitrogen, and evaluates their reactivity in Al/CuO nanothermites. High-resolution transmission electron microscopy (HR-TEM) revealed a conformal ∼4.6 nm PPh_3_-derived shell on the crystalline Al core. No distinct oxide interlayer was resolved within the spatial and contrast limits of the images. X-ray diffraction (XRD) confirmed crystalline fcc Al with no detectable Al_2_O_3_, and X-ray photoelectron spectroscopy (XPS) resolved an Al 2p component at ∼74.7 eV, consistent with an interfacial interaction between the PPh_3_-derived layer and the Al surface. Solid-state ^31^P NMR showed that the shell is a PPh_3_-derived organophosphorus layer with partial oxidation to phosphine oxide. Dynamic light scattering (DLS) gave an effective particle diameter of ∼65 nm. In air, thermogravimetric analysis-differential scanning calorimetry (TGA-DSC) showed PPh_3_ removal up to ∼450 °C followed by two-stage aluminum oxidation peaking at 631 and 880 °C. Under argon, Al@PPh_3_/CuO nanothermites exhibited a thermite onset near 543 °C, approximately 50 °C above the native oxide Al/CuO baseline. Strand burner tests in 2 mm ID capillary tubes showed a nonmonotonic burn rate dependence on Al@PPh_3_ replacement fraction: partial substitution raised the burn rate from ∼330 m/s at 0% to 387 m/s at 30%, whereas higher loadings caused a sharp decline to ∼95 m/s at 50% and prevented self-sustained propagation above 50%. The higher burn rate at 30% is attributed to a trade-off between partial removal of the native oxide diffusion barrier, augmented by shell-derived gas release, and the endothermic cost of shell decomposition at higher loadings.

## Graphical abstract

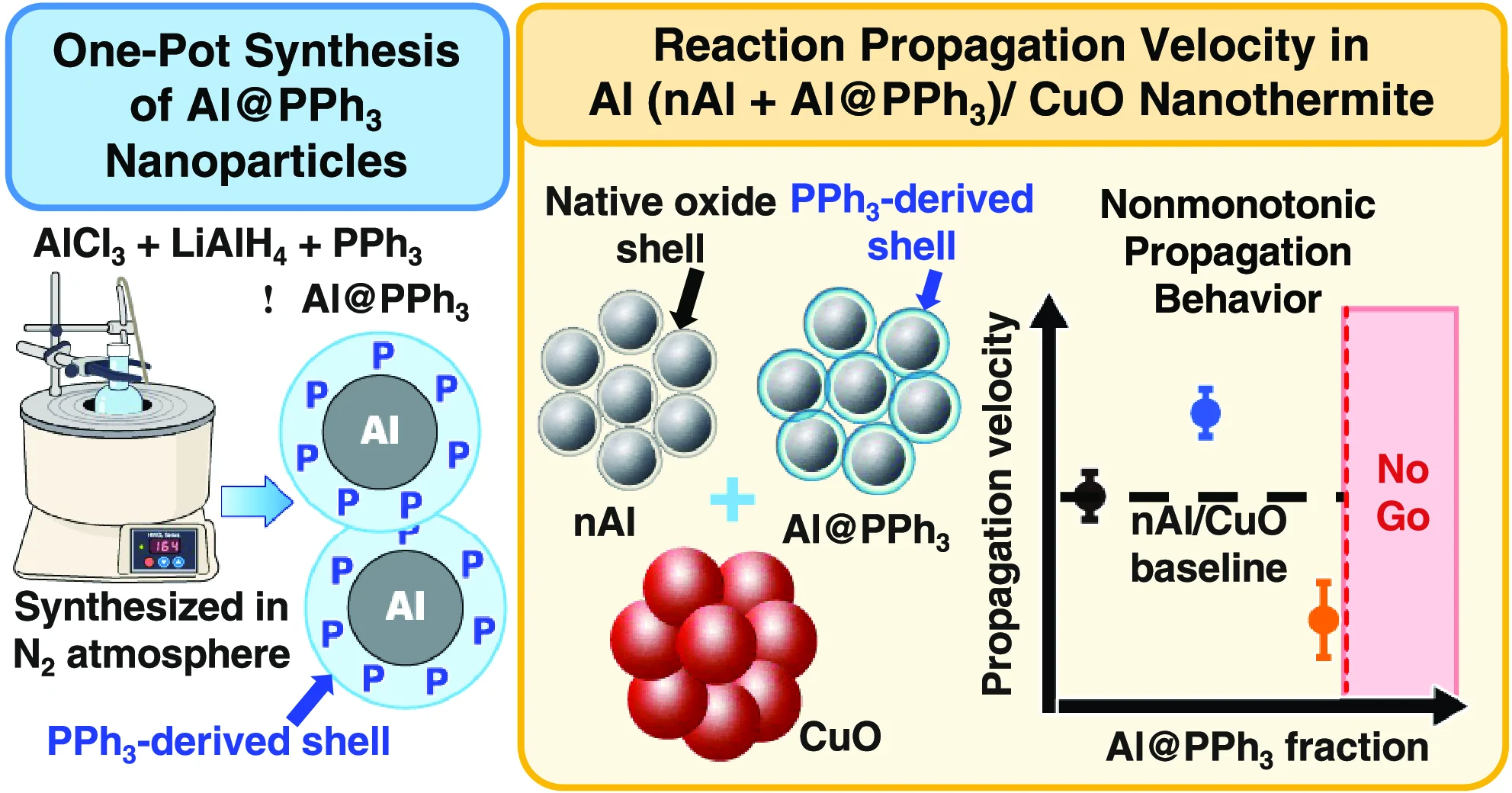

## 1 Introduction

Aluminum is one of the most widely used metal fuels in energetic applications owing to its high volumetric energy density, low cost, and relative abundance. It is used in solid rocket propellants, where it enhances specific impulse and combustion temperature.^1,2^ It is also a popular primary fuel in nanothermites, a class of metastable intermolecular composites (MICs) that pair nanoscale metal fuels with metal oxide oxidizers to achieve rapid energy release. Nanothermites have attracted attention for applications in microinitiators, primers, and on-chip energetic devices.^2–4^ However, the performance of aluminum is fundamentally limited by the native alumina (Al_2_O_3_) layer that forms spontaneously upon exposure to air or moisture. This oxide shell passivates the metallic core, slowing further oxidation during storage. But it also acts as a diffusion barrier that restricts direct contact between the aluminum and the oxidizer, thereby increasing ignition delay and reducing reactivity.^5–7^ The trade-off is particularly sensitive for nanoparticles, for which the oxide can represent a substantial fraction of the particle mass and markedly reduce the active aluminum content.^8^

The consequences of the native oxide layer are well documented across energetic applications. Trunov et al.^5^ showed that polymorphic phase transformations in the alumina influence aluminum oxidation kinetics, with the shell persisting as an inert barrier through the ignition event. In terms of their effects on reactivity, Sullivan et al.^9^ identified the reactive sintering mechanism as an important part of Al/CuO nanothermite combustion, underscoring the critical role of interfacial contact in determining reactivity. Burn rates in confined nanothermite geometries can range from a few m/s in densely pressed pellets to over 1000 m/s in loosely packed powders, a variation largely affected by gas phase convective transport through the porous bed driven by gaseous products of the thermite reaction.^1,10–12^ The passivation layer modulates burn rate through several mechanisms. It sets the active aluminum content and the ignition threshold. For organic coatings, thermal decomposition also releases gaseous species that can either augment or disrupt convective propagation, depending on the timing and volume of gas release.^1^ The choice of passivation strategy therefore directly impacts both the achievable energy output and the propagation behavior of nanothermite systems. Similarly, in propellant applications, the oxide shell represents inert mass that lowers the effective fuel loading, and the barrier it imposes on aluminum combustion contributes to incomplete oxidation and agglomerate formation.^1,13^

To address these limitations, a range of passivation strategies have been developed. These can be broadly grouped into two categories: methods that apply a functional coating over the pre-existing native oxide layer, and one-pot synthesis approaches that simultaneously generate and passivate aluminum nanoparticles, minimizing native oxide formation during the synthesis.^14,15^ Among coating-based strategies, reactive metallic coatings such as nickel trigger exothermic intermetallic reactions with aluminum well below the oxide-mediated ignition threshold.^16^ Metal oxide coatings can serve as both a passivation layer and an intimate oxidizer source. For example, Mn_3_O_4_ coatings enhance the heat release associated with the thermite reaction at the particle surface,^17^ while atomic layer deposited CuO nanocoatings offer precise control over energy release behavior.^18^ Fluoropolymer coatings, such as PTFE and PVDF, undergo preignition fluorination reactions with aluminum, releasing additional energy and lowering ignition thresholds,^19,20^ and a recent comparative study demonstrated that fluorocarbon-capped aluminum nanoparticles exhibit superior combustion performance relative to hydrocarbon-capped counterparts, underscoring the importance of cap chemistry.^21^ Our group has also recently synthesized trifluoroacetic acid (TFA) encapsulated aluminum nanoparticles and characterized their Al/CuO nanothermite reaction performance.^22^

Nonreactive organic coatings offer a different trade-off: oleic acid, waxes, and stearic acid provide hydrophobic protection and long-term storage stability, decomposing at relatively low temperatures to expose the aluminum core prior to ignition.^8,23^ Other approaches include epoxide-based capping with 1,2-epoxydodecane, which mitigates oxide formation during synthesis and yields active Al fractions up to ∼94% of total Al content,^24^ and perfluoroalkyl carboxylic acids bonded directly onto bare nanoparticle surfaces as self-assembled monolayers.^25^ Surface modification via fluoroalkylsilane coupling agents^26^ and various nonoxide coatings of different chemical origins^27^ represent additional variations. Recent advances have further shown that polydopamine/polyethylenimine nanocoatings significantly enhance both stability and combustion performance,^28^ while nonthermal hydrogen plasma treatment offers a solvent-free approach to engineer surface chemistry for improved energetic output.^29^ However, the majority of these strategies, including oleic/stearic acids, fluoroalkylsilanes, polydopamine/PEI, and plasma-modified coatings, are applied on top of a pre-existing native oxide. In these cases, the oxide layer remains between the aluminum core and the functional coating, contributing inert mass and imposing a persistent diffusion barrier. Only a small subset of approaches, such as perfluoroalkyl carboxylic acid self-assembled monolayers on bare Al^25^ and epoxide polymerization during aluminum particle synthesis,^24^ achieve an oxide-free or oxide-minimized interface.

Triphenylphosphine (PPh_3_) is a promising candidate for addressing this limitation. It is introduced into the reaction medium before reduction rather than applied over a pre-existing oxide, so the organic layer forms on aluminum generated in situ under an inert atmosphere. Cui et al. used this route to prepare Al@PPh_3_ nanoparticles and reported shell thicknesses in the 2–6 nm range.^14,30^ Two features make such particles of interest for energetic formulations. First, because the shell is established during particle formation, less native oxide is expected to be incorporated at the core–shell interface than for coatings applied after synthesis. In addition, the PPh_3_-derived shell volatilizes and decomposes at temperatures well below typical nanothermite ignition thresholds (∼400 °C versus ∼550 °C for Al/CuO), so the protective layer is largely removed before the main exothermic reaction initiates. This contrasts with fluoropolymer coatings that actively participate in preignition fluorination reactions,^19^ and with native oxide shells that persist as diffusion barriers through the ignition event. The bulky triphenyl groups are also expected to provide steric and hydrophobic protection during handling and storage.

What has not been established is how such particles behave in an energetic formulation. Previous studies employing PPh_3_ as a passivation agent have focused on the synthesis and characterization of Al@PPh_3_ nanoparticles for nonenergetic applications such as solar cells and catalysis.^14^ Neither the reactivity nor the propagation behavior of PPh_3_-passivated aluminum has been examined in nanothermites or propellants. The outcome is not predictable from the coating chemistry alone, because an organic shell contributes inert mass and an endothermic decomposition load in addition to any benefit from reduced oxide content. Whether PPh_3_ passivation is a net benefit or a net penalty for combustion performance is therefore an open question, and it is the question this work addresses.

This work addresses that question in three steps. Al@PPh_3_ nanoparticles were first synthesized at a PPh_3_/AlCl_3_ molar ratio of 1:1, an order of magnitude below the ratios used previously,^30^ in order to limit the organic content that the energetic application must carry. The composition of the resulting surface layer was then determined by scanning electron microscopy (SEM), dynamic light scattering (DLS), high-resolution transmission electron microscopy (HR-TEM), X-ray diffraction (XRD), Fourier-transform infrared (FTIR), X-ray photoelectron spectroscopy (XPS), solid-state ^31^P MAS NMR, and thermogravimetric analysis-differential scanning calorimetry (TGA-DSC). Finally, the particles were evaluated in Al@PPh_3_/CuO nanothermites by thermal analysis at several equivalence ratios and by capillary tube strand burner tests at varying replacement fractions of commercial oxide-passivated aluminum. The solid–solid nanothermite configuration was chosen for the reactivity assessment because propagation in this configuration depends on condensed phase fuel-oxidizer contact and heat feedback within the packed bed instead of simple exposure to gas phase oxygen, which makes it a stringent test of whether a modified fuel particle delivers a net benefit or a net energetic penalty.

## 2 Experimental Section

### 2.1 Synthesis of Al@PPh_3_ Nanoparticles

Aluminum nanoparticles were synthesized via a wet chemical reduction process using aluminum chloride (AlCl_3_, 98% Sigma-Aldrich) and lithium aluminum hydride (LiAlH_4_, 99% Sigma-Aldrich) as precursors in mesitylene (1,3,5-trimethylbenzene, 99% Sigma-Aldrich). The procedure was adapted from the wet chemical method reported by Cui et al.^14,30^ All precursor preparation was conducted in a glovebox under a nitrogen atmosphere. AlCl_3_ (20 mmol) and PPh_3_ (20 mmol, 99%, Sigma-Aldrich) were first dispersed in 40 mL mesitylene and stirred for 0.5 h, after which a 10 mL mesitylene solution containing LiAlH_4_ (60 mmol) was slowly added under continuous stirring.

The initial hydride transfer reaction between AlCl_3_ and LiAlH_4_ is expected to produce AlH_3_ or related aluminum hydride intermediates together with LiCl. Under the reflux conditions employed in this study, these thermally unstable hydride species decompose to metallic Al and H_2_. The resulting Al atoms subsequently nucleate and grow into nanoparticles, while PPh_3_ present in the reaction medium limits particle growth and aggregation. The overall reaction may be represented as 3LiAlH_4_ + AlCl_3_ → 4Al + 3LiCl + 6H_2_, although the actual solution phase process may involve several hydride-containing intermediates.

A schematic of the overall synthesis process is shown in Figure 1. The precursor solution was heated to 164 ± 1 °C and maintained for 1 h under nitrogen atmosphere. This temperature corresponds to the boiling point of mesitylene and ensures complete reduction of the aluminum precursors. Under these conditions, the refluxing solvent provides uniform heating while the nitrogen blanket prevents premature oxidation of the incipient aluminum particles. This “one-pot” procedure in an oxygen-free environment minimizes the potential of native oxide formation on the aluminum particles. The synthesized Al@PPh_3_ nanoparticles were collected by centrifugation at 12,000 rpm for 10 min at −10 °C to improve collection yield, washed three times with methanol (99.8% Fisher) to remove unreacted precursors and residual mesitylene, and vacuum-dried at 110 °C for 24 h. A PPh_3_/AlCl_3_ molar ratio of 1:1 was used in this work in contrast to the substantially higher ratios (15:1 and 20:1) reported in the literature.^30^ The present ratio provides sufficient PPh_3_ surface coverage to form a continuous protective shell on the aluminum nuclei, while the formation of a stable PPh_3_-AlCl_3_ adduct moderates the precursor reduction kinetics during synthesis. The reduced ratio also limits the organic content of the product, an important consideration for the energetic application, since excess organic material adds inert mass and an endothermic decomposition load that can suppress or completely inhibit combustion (Section 3.4).

**Figure 1:**
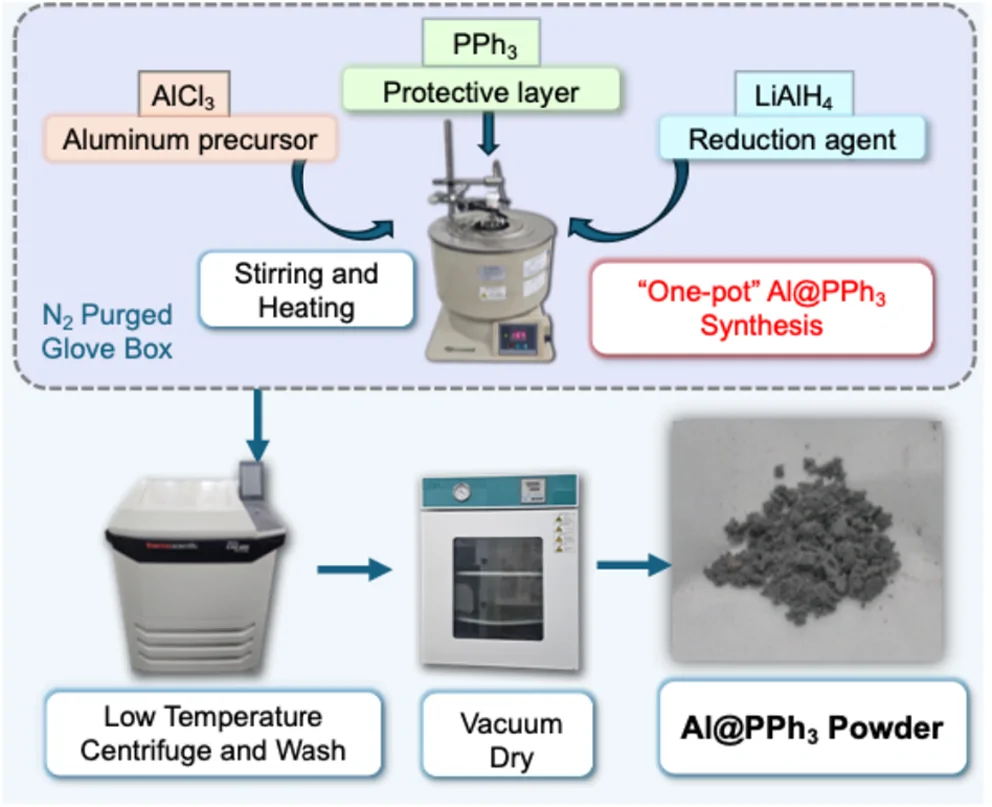
Schematic of the one-pot synthesis process for Al@PPh_3_ nanoparticles.

### 2.2 Preparation of Nanothermite Powders

The nanothermite composites were prepared via physical blending. The constituent materials included the Al@PPh_3_ nanoparticles synthesized in-house, commercial 50 nm aluminum powder with native Al_2_O_3_ shell (Bojun Co. Ltd.), and 80 nm CuO powder (US Research Nanomaterials, Inc.). The nanopowders were dispersed in hexane, ultrasonicated to ensure homogeneous mixing, and subsequently vacuum-dried. The equivalence ratio (ϕ_Al_) was defined based on the stoichiometric reaction between aluminum and CuO, considering only the aluminum core as the fuel. The PPh_3_ organic layer was excluded from the fuel mass in this calculation since it may not participate in the thermite reaction. The composition ratios refer to the mass fraction of Al@PPh_3_ in the binary mixture of Al@PPh_3_ and commercial aluminum powder, ranging from 0% to 100%.

The desired masses of Al@PPh_3_, commercial Al, and CuO powders were weighed according to the target equivalence ratio and Al@PPh_3_ fraction. The powders were added to hexane in a glass beaker and dispersed using an ultrasonic probe to break up agglomerates. The beaker was placed in an ice bath, and 10 s on/10 s off intermittent ultrasonic cycles were applied to avoid excessive heating. The homogenized suspension was subsequently transferred to a vacuum oven and dried at 60 °C for 12 h to remove the hexane, yielding dry nanothermite powder.

### 2.3 Characterization

Particle morphology was examined using scanning electron microscopy (SEM), while HR-TEM provided insight into the core–shell structure and coating thickness. XRD analysis was conducted to identify the crystalline phases present in the synthesized nanoparticles. Solid-state ^31^P NMR spectra of Al@PPh_3_ were acquired at 9.4 T (Avance III HD 400 MHz, Bruker). Crystalline PPh_3_ (99%, Sigma-Aldrich) was measured under identical conditions as a reference. Thermal properties, including decomposition and oxidation behavior, were evaluated using TGA-DSC (SDT Q600, TA Instruments). Measurements were performed over a temperature range of 30–1000 °C at a heating rate of 10 °C/min. Al@PPh_3_ nanoparticles were characterized in air to evaluate oxidation behavior, while nanothermite mixtures were analyzed under argon to isolate the thermite reaction from atmospheric oxidation. The purge gas flow rate was 100 sccm in all cases.

### 2.4 Burn Rate Tests

Strand burner tests were conducted to evaluate the burning velocity of thermite composites with varying proportions of Al@PPh_3_ and commercial aluminum powder. The prepared thermite powders were loaded into 10 cm long borosilicate tubes with an inner diameter of 2 mm and ignited using an electric match. Samples were placed inside a chamber with sight windows. The combustion process was recorded using a color high-speed camera (MiroLab310, Vision Research). The nanothermite powder was loaded by alternately filling small portions into the tube and tapping the tube vertically against a hard surface to consolidate the powder. This fill-and-tap procedure was repeated until the tube was filled to the desired length. Packing density of the filling was 1.05 ± 0.15 g/cm^3^. Experiments were carried out at ambient pressure and temperature. Three repeated tests were performed per condition. Cases in which self-sustained reaction propagation throughout the tube could not be established, including those with a short initial propagation followed by extinction, were classified as failure (No Go). The burn rate was determined from the high-speed video by tracking the luminous flame front position over time along the tube axis. The average propagation velocity was calculated from a linear fit to the flame front position versus time data over the steady-state portion of the burn. Error bars in the burn rate figures represent the standard deviation from three repeated tests.

## 3 Results and Discussion

### 3.1 Morphological, Crystalline, and Surface Characterization

SEM images of the synthesized Al@PPh_3_ nanoparticles (Figure 2) reveal roughly spherical particles with diameters predominantly in the range of 40–60 nm. Particle agglomeration is evident in the micrographs, consistent with the high surface energy of metallic nanoparticles synthesized by wet chemical methods, where interparticle van der Waals forces promote clustering during the drying step. Dynamic light scattering measurements (Brookhaven ZetaPALS) of particles redispersed in solution, shown in Figure 3, yielded a narrow, approximately Gaussian size distribution centered near 65 nm, with a mean diameter of 74.6 nm, an intensity-weighted effective diameter of 65.3 nm, and a standard deviation of 7.5 nm. The slight upward shift relative to the SEM observations likely reflects contributions from the hydrodynamic envelope of the PPh_3_-derived shell and residual clustering of particles in suspension.

**Figure 2:**
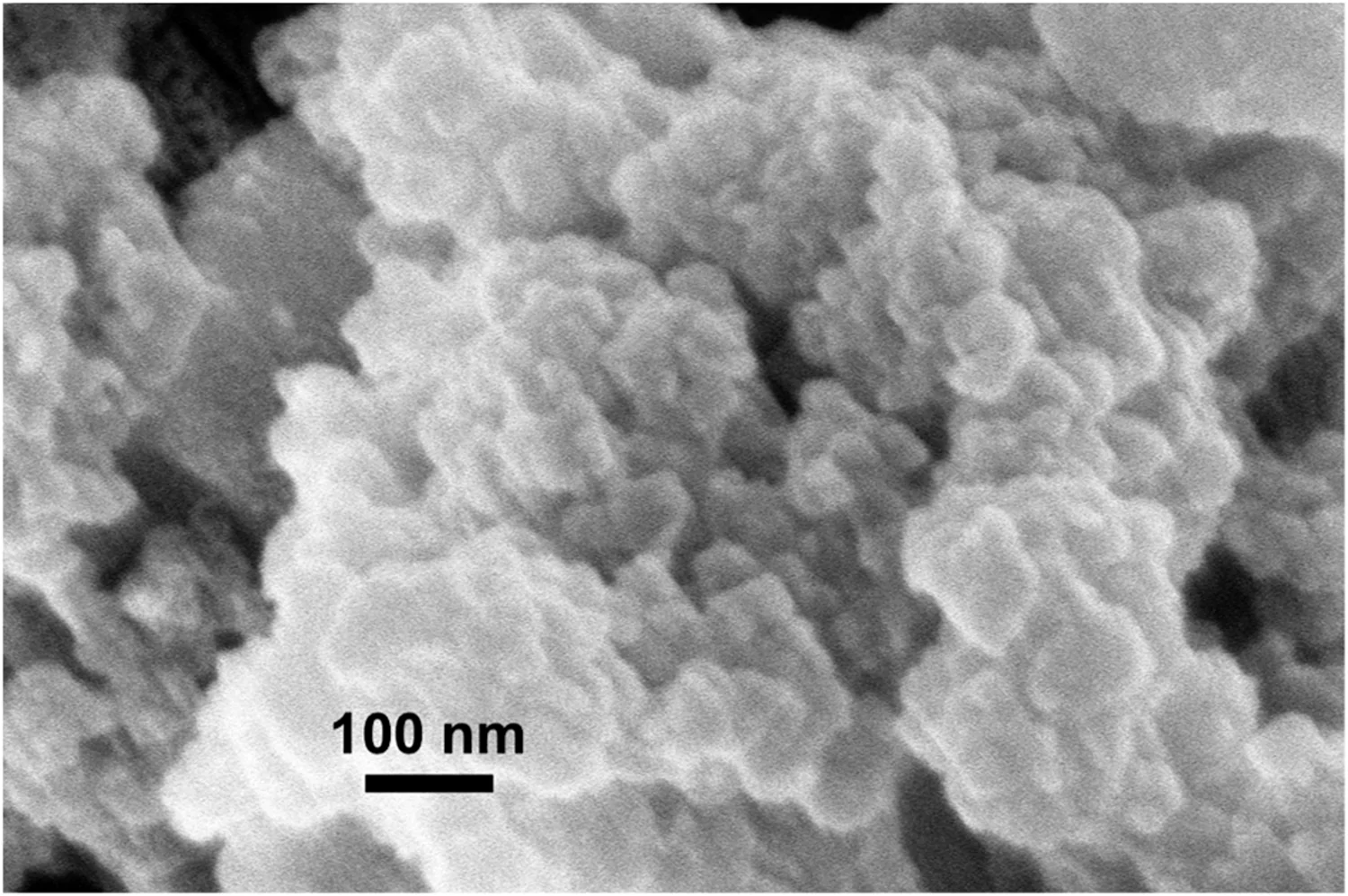
SEM images of Al@PPh_3_ nanoparticles synthesized at PPh_3_/AlCl_3_ = 1:1.

**Figure 3:**
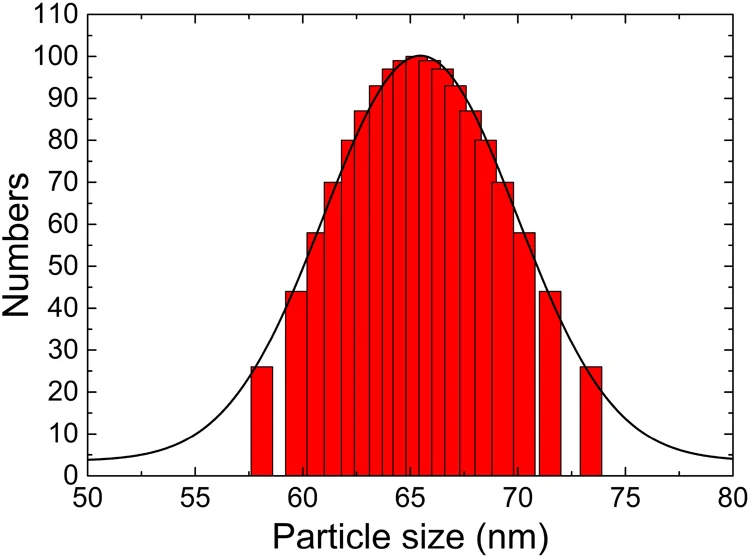
Particle size distribution of Al@PPh_3_ nanoparticles from dynamic light scattering.

HR-TEM analysis revealed a conformal and uniform PPh_3_ coating layer surrounding the aluminum core, indicating successful formation of a core–shell structure (Figure 4). The coating layer appears amorphous and continuous, consistent with the noncrystalline nature of PPh_3_ and similar to organic coatings reported in prior studies.^24^ For nanoparticles synthesized at a PPh_3_/AlCl_3_ ratio of 1:1, the coating thickness is approximately 4.6 nm. The HR-TEM images show no resolvable oxide interlayer between the aluminum core and the PPh_3_-derived layer within the spatial and contrast limits of the images, although a very thin amorphous Al–O layer below these limits cannot be excluded. Any such oxide would most likely originate from oxidation of incompletely capped surface regions during methanol washing, transfer, or storage rather than from growth during the oxygen-free synthesis and would therefore be expected to be thin and discontinuous rather than a continuous native shell. This contrasts with commercially available aluminum nanoparticles, where a 2–4 nm amorphous Al_2_O_3_ shell is invariably present.^5^ These coating thicknesses are comparable to those reported for other organic passivation systems. Jouet et al.^25^ achieved 1–3 nm perfluoroalkyl coatings, while Cui et al.^14^ reported PPh_3_ layers in the 2–6 nm range depending on synthesis conditions.

**Figure 4:**
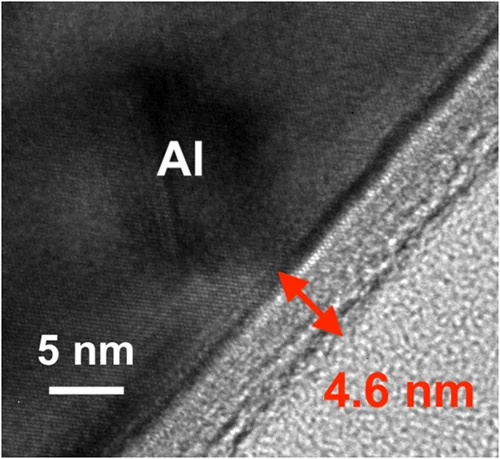
HR-TEM image of an Al@PPh_3_ nanoparticle showing the core–shell structure.

XRD analysis of the synthesized nanoparticles (Figure 5) confirmed the presence of crystalline metallic aluminum peaks with no detectable aluminum oxide signals, further supporting the successful passivation of the aluminum particles with PPh_3_ while avoiding surface oxidation during synthesis. We note that XRD primarily excludes a crystalline Al_2_O_3_ layer; the absence of a thin amorphous oxide envelope is inferred from the HR-TEM observations together with the TGA mass balance presented in Section 3.2. The diffraction pattern shows characteristic face-centered cubic (fcc) aluminum peaks at 2θ ≈ 38.5°, 44.7°, 65.1°, and 78.2°, corresponding to the (111), (200), (220), and (311) planes, respectively. The absence of α-Al_2_O_3_ or γ-Al_2_O_3_ reflections indicates that no crystalline oxide phase formed during synthesis, although it does not rule out a thin amorphous oxide.

**Figure 5:**
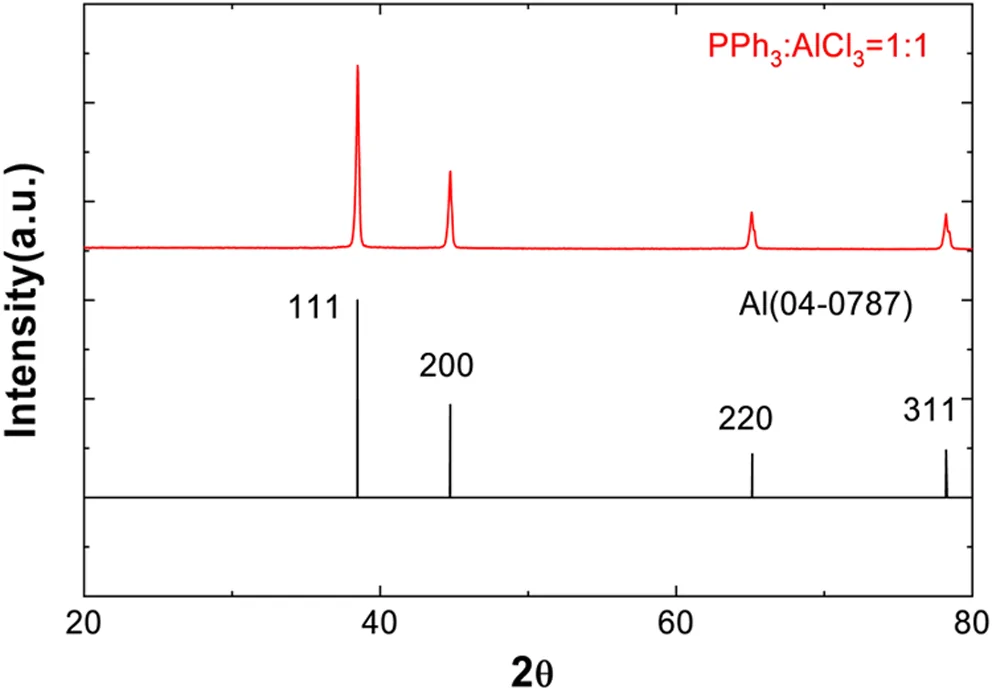
XRD pattern of the synthesized Al@PPh_3_ nanoparticles.

The FTIR spectra of the Al@PPh_3_ nanoparticles shown in Figure 6 exhibit several characteristic absorption bands associated with the presence of PPh_3_-derived species on the nanoparticle surface. Although the aromatic C–H vibrational modes near 3001 cm^–1^ and 742 cm^–1^ are not clearly distinguishable, as they are embedded within stronger overlapping absorptions, the characteristic PPh_3_ features remain readily discernible: bands at ∼1475 cm^–1^ and ∼1435 cm^–1^ correspond to benzene ring skeletal vibrations, and a distinct feature at ∼1079 cm^–1^ is assigned to P–C bonding. Together, these absorptions provide clear evidence for the presence of a PPh_3_-derived organic layer on the nanoparticle surface. Furthermore, a broad absorption band in the range of 3500–3000 cm^–1^ is observed, which can be attributed to O–H stretching vibrations, probably from the methanol and moisture trapped in the nanoparticle cluster. The accompanying H–O–H bending band at ∼1630 cm^–1^ confirms the presence of adsorbed water. Such residual oxygen-containing species are difficult to remove completely even after vacuum drying and may also contribute to oxygen related signals in surface sensitive measurements.

**Figure 6:**
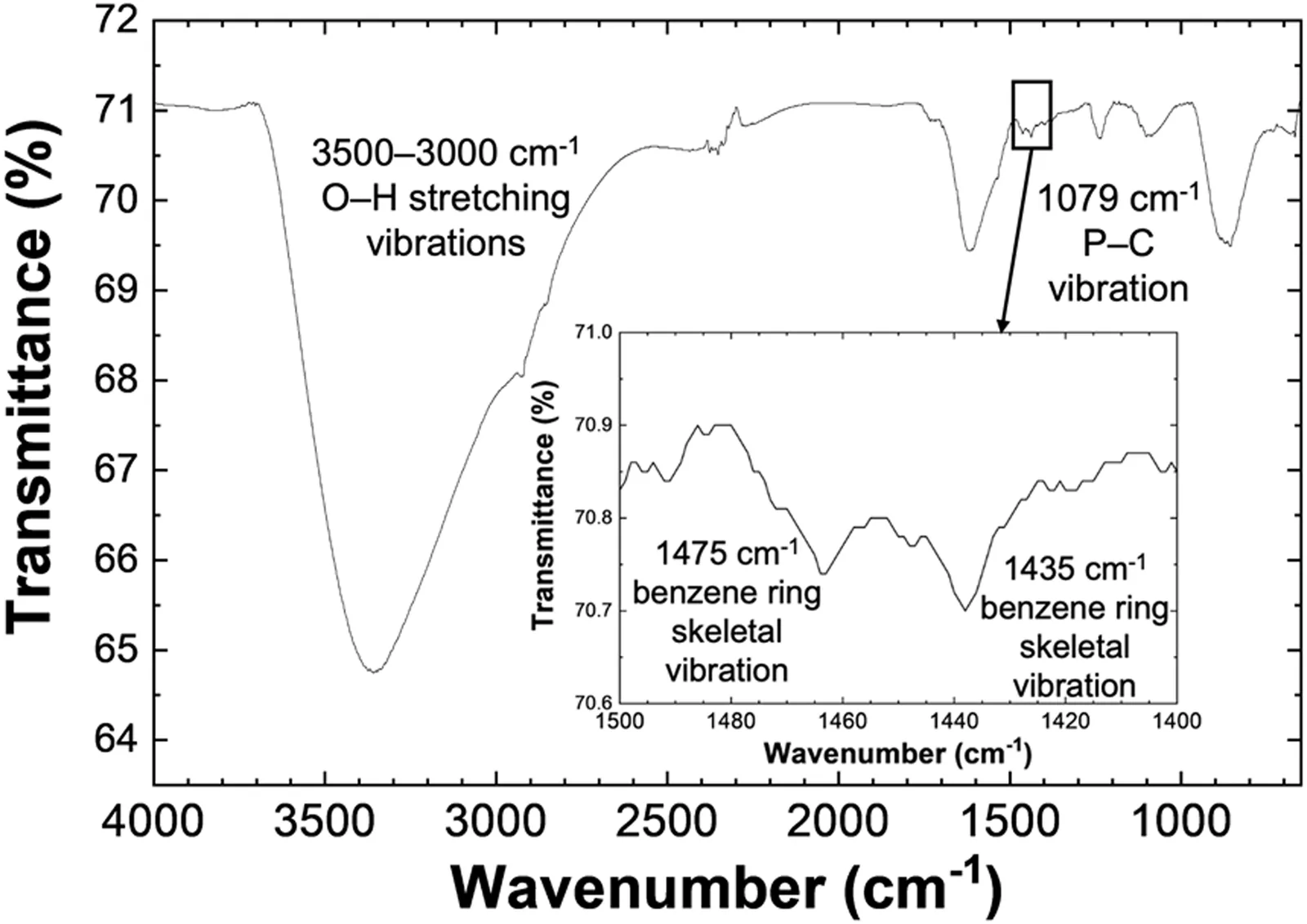
FTIR spectrum of the Al@PPh_3_ particles showing the absorption peaks of the PPh_3_-derived layer.

XPS was employed to investigate the surface chemical composition of the Al@PPh_3_ nanoparticles, with the spectrum shown in Figure 7. Resolving the phosphorus signal in PPh_3_-passivated aluminum nanoparticles is inherently difficult because uncoordinated PPh_3_ typically shows a P 2p peak at ∼130–131 eV, but in aluminum-rich nanoparticles, the nearby Al 2s signal at ∼118–121 eV produces a strong neighboring background that suppresses the visibility of the weak P 2p region features. This effect becomes even more pronounced as the PPh_3_-derived surface layer is thin and the surface phosphorus concentration is low. Therefore, the absence of a clearly resolved P 2p peak does not preclude the presence of phosphorus-containing ligands, which is independently supported by the FTIR results.

**Figure 7:**
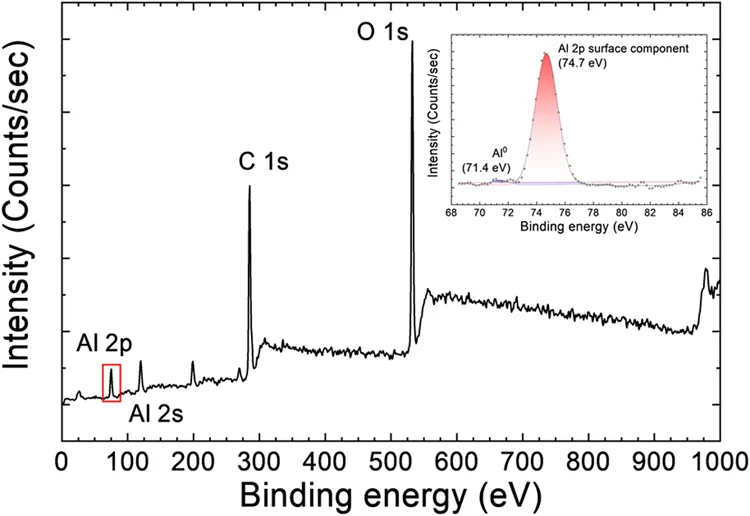
XPS spectrum of the Al@PPh_3_ particles.

Diagnostic evidence is instead obtained from the Al 2p region (insert of Figure 7). A peak centered at ∼74.7 eV was observed in the Al 2p region. This feature is consistent with a chemically modified Al surface and may include contributions from interfacial Al–P-containing species as well as Al–O species formed during post synthesis handling, which overlap in this binding energy range. After curve fitting, a weak feature near 71.4 eV, attributable to the metallic Al core, is discernible. The low intensity is expected from the surface sensitivity of XPS. With an inelastic mean free path of a few nanometers, photoelectrons from the metallic core are strongly attenuated by the ∼4.6 nm PPh_3_-derived shell. The coexistence of these two components supports the presence of a modified surface layer while confirming that the underlying metallic Al phase is retained.

Solid-state ^31^P MAS NMR identifies the phosphorus environments present in the surface layer. Figure 8 compares Al@PPh_3_ with crystalline PPh_3_ recorded under identical conditions. The reference gives a single narrow resonance at −9.0 ppm. In Al@PPh_3_ that resonance is absent, so the ligand does not survive the synthesis and workup. Two downfield components appear instead, at 21.1 and 34.8 ppm. The 34.8 ppm component is assigned to a triarylphosphine oxide (Ar_3_P=O), in agreement with reported values for aryl phosphine solids^31^ and with the 1179 cm^–1^ P=O band in the FTIR spectrum (Figure 6). The 21.1 ppm component is a second organophosphorus environment that cannot be assigned from its chemical shift alone. The surface layer is therefore a substantially oxidized PPh_3_-derived organophosphorus layer rather than intact, coordinated PPh_3_. A more detailed study of the interfacial bonding and surface composition is left to future work.

**Figure 8:**
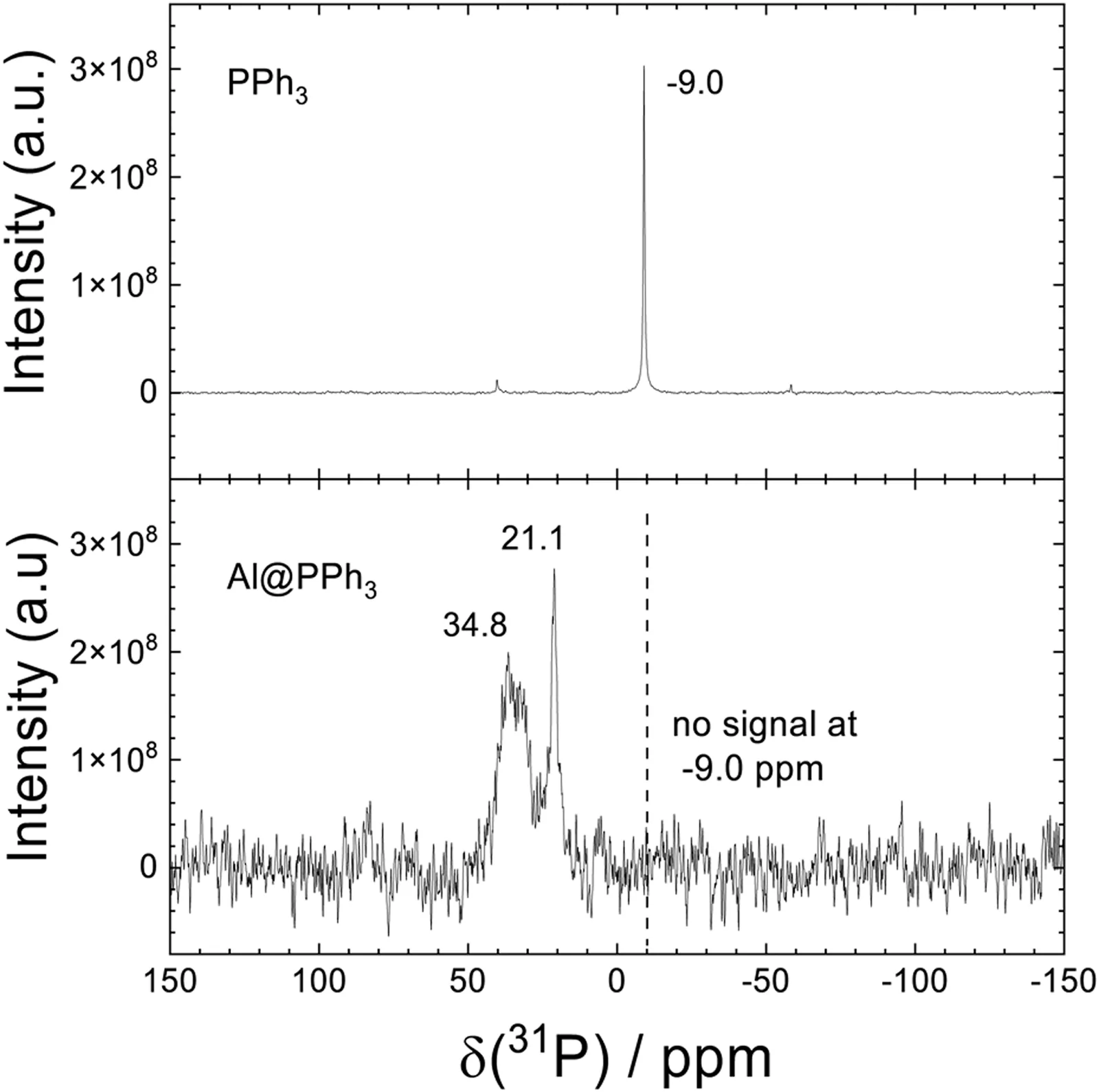
Solid-state ^31^P NMR spectra of crystalline PPh_3_ and Al@PPh_3_, both recorded at a spinning rate of 8 kHz.

### 3.2 Thermal Analysis of Al@PPh_3_ Nanoparticles

TGA-DSC analysis of the synthesized Al@PPh_3_ nanoparticles was performed in air at a heating rate of 10 °C/min, and the results are shown in Figure 9 along with the corresponding DTG trace. The TG curve shows a continuous mass loss from room temperature to ∼540 °C (total ∼ 21 wt %), reaching a minimum of ∼79 wt %, followed by two distinct mass gain steps that bring the final mass to ∼110 wt % at 1000 °C, the upper limit of the measurements. The DTG trace shows two negative valleys with minima at 95 and 236 °C during the mass loss regime, then returns toward zero between ∼250 and ∼540 °C, crosses baseline and exhibits two sharp positive peaks at 631 and 880 °C that coincide with the TG mass gain steps. The DSC trace shows a broad endotherm overlapping the first two DTG valleys, followed by a small exothermic peak near 500 °C (visible in the inset), a return toward baseline with a slight minimum at 540 °C, and two major exothermic peaks: the first spanning 551–683 °C, and the second spanning 759–980 °C. These peak positions align with the two DTG peaks. A discontinuity at 656 °C is evident on the descending shoulder of the first DSC peak.

**Figure 9:**
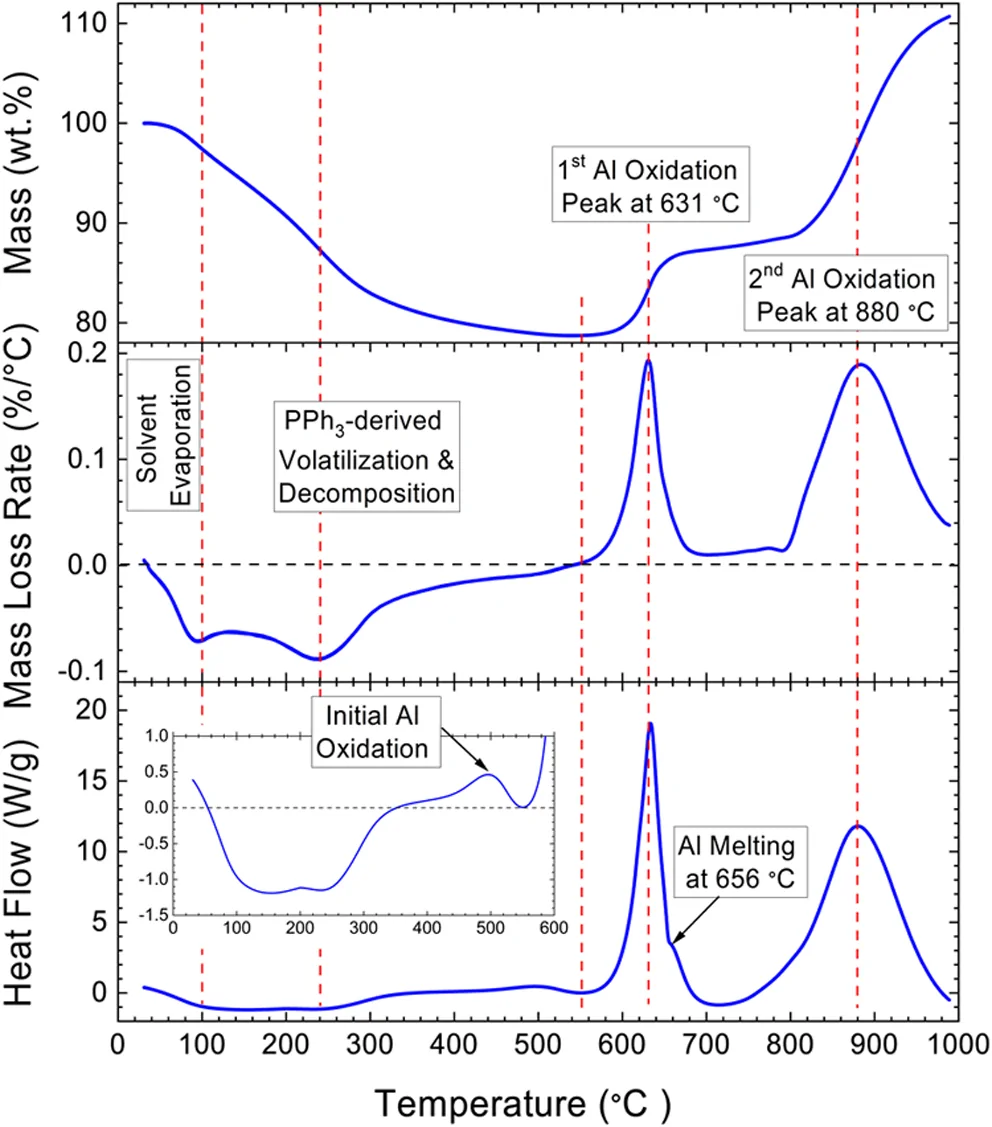
TGA-DSC curves of Al@PPh_3_ nanoparticles in air at 10 °C/min, with a small exothermic peak near 500 °C, visible in the inset.

The integrated heat release is 4003 J/g for the first-peak and 7266 J/g for the second. The first-peak heat release is comparable to the 3920 J/g reported by Guo et al.^23^ for fresh 50 nm Al nano powders with a thin native oxide layer, and consistent with the expected benefit of the PPh_3_-derived shell. The organic coating minimizes the formation of the oxide interface on the Al core during synthesis and storage, thereby allowing a larger fraction of the aluminum to remain metallic and contributing to the oxidation exotherm during the TGA measurement. This comparison indicates that the one-pot synthesis successfully produces Al nanoparticles with an intact metallic core protected by the organic shell.

The features on TG-DTG-DSC curves correspond to a sequence of physical and chemical processes. The DTG valley at 95 °C and accompanying endotherm are attributed to evaporation of residual solvent (mesitylene and methanol) trapped within particle agglomerates, accounting for ∼2 wt % of the initial mass. The DTG valley at 236 °C represents volatilization of the majority of the PPh_3_-derived shell. The endothermic signature and broad temperature range are consistent with physical volatilization of the loosely bound outer PPh_3_ layers. Between ∼250 and ∼450 °C, the DTG returns gradually toward zero while the DSC shifts from endothermic to mildly exothermic, indicating that the remaining PPh_3_ is removed through oxidative decomposition in the air sweep. The total mass loss attributable to PPh_3_ removal is ∼19 wt %, consistent with the ∼4.6 nm shell thickness and ∼50 nm core diameter. The small exothermic peak near 500 °C marks the onset of aluminum oxidation as the exposed core reacts with oxygen, potentially forming a thin amorphous Al_2_O_3_ layer that rapidly repassivates the surface. This thin oxide is below the detection limit of the TG curve but is evident in the DTG trace, which trends continuously upward through this window as oxide formation gradually offsets residual organic loss.

The DSC onset of rapid exothermic reaction at 551 °C coincides with the DTG crossing baseline near 540 °C, indicating that the thin oxide barrier can no longer contain the accelerating oxidation. The first DTG and DSC peaks at 631 °C represent the main oxidation of the aluminum core. The discontinuity at 656 °C corresponds to melting of the aluminum core. Between ∼700 and ∼790 °C, the DSC minimum at 713 °C reflects a pause in oxidation as the thickened γ-Al_2_O_3_ shell formed during the first oxidation blocks further reaction. The second oxidation event peaks at 880 °C and is broader and more gradual, reflecting the increasingly diffusion-controlled nature of oxidation through the progressively thickening oxide layer. This two-stage oxidation pattern is consistent with the polymorphic phase transformation mechanism established for micron-sized aluminum^5,32^ and confirmed to follow a qualitatively similar sequence in nanoparticles.^32,33^ The second event corresponds to the γ-Al_2_O_3_ → θ-Al_2_O_3_ transformation, whose accompanying volume contraction disrupts the shell and opens new diffusion paths for oxygen to reach the molten aluminum core.

### 3.3 Thermal Analysis of Al@PPh_3_/CuO Nanothermites

TGA-DSC analysis of Al@PPh_3_/CuO nanothermites at three equivalence ratios (ϕ_Al_ = 0.8, 1.0, and 1.6) and a reference Al/CuO nanothermite with native oxide passivation (ϕ_Al_ = 1.0) was performed under argon at a heating rate of 10 °C/min, and the results are shown in Figure 10. For the native oxide Al/CuO sample, the TG curve remains relatively flat across the tested temperature range, with less than 1% variation. The corresponding DTG trace is noisy at this scale and should not be overinterpreted. The DSC trace, however, exhibits a sharp, intense exothermic peak with onset around 490 °C and peak at 567 °C, followed by a smaller exothermic peak extending up to 850 °C. The second peak is difficult to quantify due to the significant baseline drift and overlapping with the release of oxygen through thermal decomposition of CuO to Cu_2_O,^34^ which is typical for TGA-DSC measurements of Al/CuO nanothermites with native oxide passivation.

**Figure 10:**
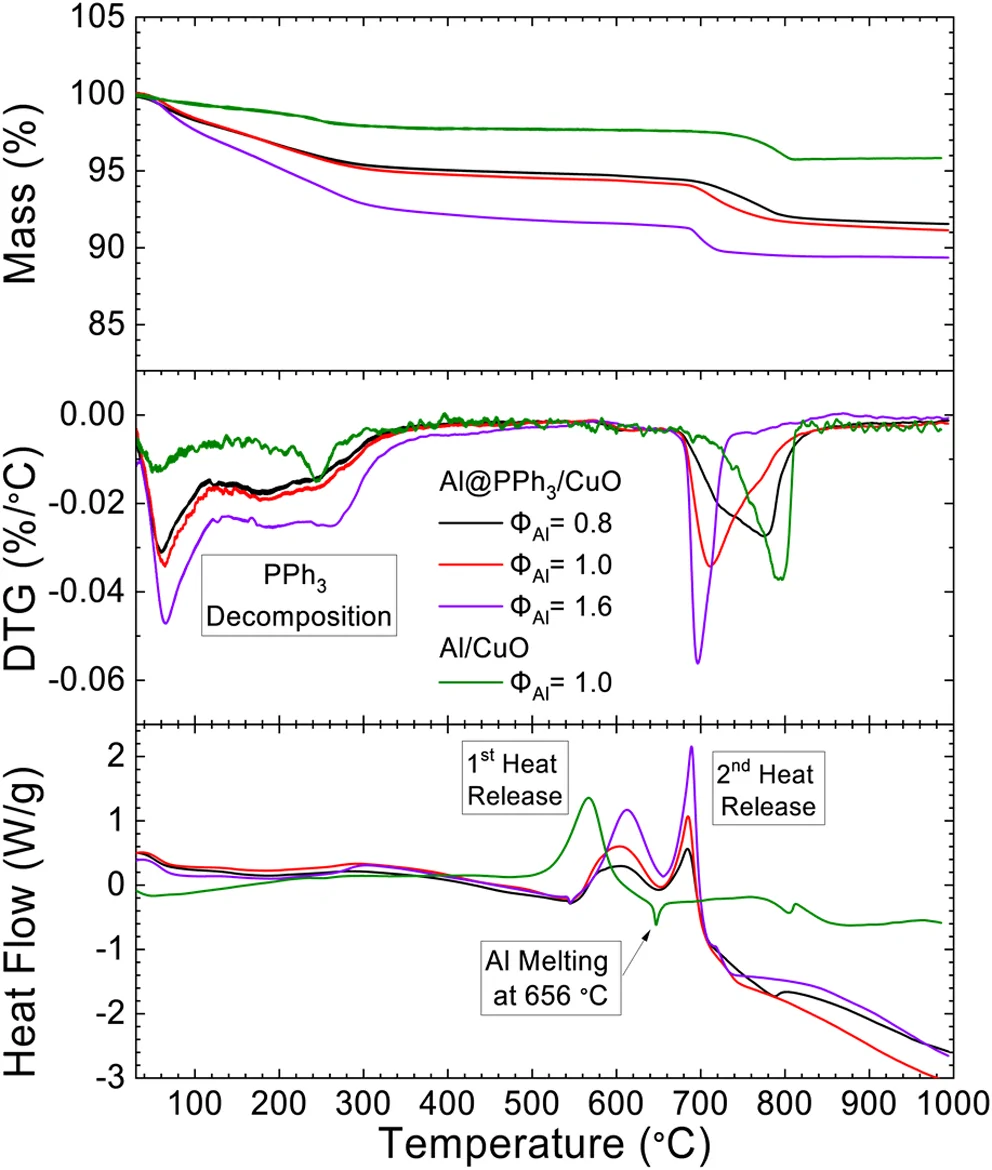
TGA-DSC and DTG curves of Al@PPh_3_/CuO nanothermites at ϕ_Al_ = 0.8, 1.0, and 1.6.

The Al@PPh_3_/CuO samples exhibit distinctly different behavior. The TG curves show a gradual mass loss from room temperature to ∼300 °C, followed by a near-flat plateau. This mass loss is reflected in the DTG traces, which display a broad negative trough below ∼270 °C that returns to near zero at ∼330 °C. The magnitude of the initial loss increases with equivalence ratio; the Al@PPh_3_-rich sample (ϕ_Al_ = 1.6) shows the largest total mass loss, as it contains the most PPh_3_. A second sharp mass drop occurs near 690 °C and levels off beyond ∼790 °C, likely associated with oxygen release from CuO dissociation under the low oxygen partial pressure condition discussed above. This loss appears as a sharp negative spike between 700 and 770 °C on the DTG traces, which shifts to a lower temperature in the richer mixtures.

In the DSC traces, an endothermic valley at ∼660 °C, corresponding to Al melting, is observed in every sample. In the Al@PPh_3_/CuO samples, the two exothermic peaks flanking this melting valley, representing the solid–solid thermite reaction and the subsequent reaction between molten aluminum and the oxides, respectively, are connected, forming a nearly continuous exothermic envelope. In the native oxide Al/CuO sample, by contrast, these peaks are separated by a wider interval, and the second heat-release peak is relatively flat. No comparable high-temperature exothermic peak is observed in the Al@PPh_3_/CuO samples within the measuring range. Among the three equivalence ratios, the Al-rich sample (ϕ_Al_ = 1.6) shows the largest heat release. The first peaks of Al@PPh_3_/CuO samples have an onset near 543 °C, approximately 50 °C higher than that of the native oxide Al/CuO. The peaks reach their maximum at around 610 °C, compared to 567 °C for the Al/CuO baseline. For ϕ_Al_ = 1.0, the integrated heat release is ∼552 J/g for the first-peak and ∼438 J/g for the connected second peak, giving a total of ∼990 J/g for the Al@PPh_3_/CuO system, higher than the ∼560 J/g first-peak release of the native oxide Al/CuO reference. It should be noted, however, that the integrated values may not represent the exact heat release, as the baseline of the DSC curves is distorted after the first heat release.

The gradual mass loss below ∼300 °C in the Al@PPh_3_/CuO samples is attributed to volatilization and decomposition of the PPh_3_-derived shell, consistent with the behavior observed in the air atmosphere TGA of Al@PPh_3_ alone (Figure 9). Under thermal analyses of nanothermite samples in argon, no atmospheric oxygen is available to repassivate the aluminum surface after PPh_3_ removal. The only oxygen source is CuO decomposition, which is limited at these temperatures. Rather than driving an immediate thermite reaction, the potential reaction scenario is that this small oxygen supply first forms a thin in situ amorphous Al_2_O_3_ layer on the exposed aluminum surface. Because the oxygen available from CuO is far more limited than atmospheric air, this layer likely remains below the critical thickness (∼4 nm) required to trigger the amorphous to γ-Al_2_O_3_ transformation that initiates rapid oxidation in the native oxide system.^5^ The thin amorphous layer therefore remains continuous and acts as a diffusion barrier, retarding further thermite reaction.

In the native oxide Al/CuO system, the pre-existing oxide shell has already reached the critical thickness by ∼490 °C. The polymorphic transformation creates cracks that allow sudden oxygen access from decomposing CuO to the aluminum core, producing the sharp, intense first exothermic peak at 567 °C. The released heat accelerates further CuO decomposition, sustaining a self-reinforcing reaction loop. As the aluminum core melts at ∼660 °C, the endothermic fusion absorbs heat and the reaction subsides. A second, broader and flatter exothermic event extends up to 850 °C, reflecting continued diffusion-controlled oxidation of the remaining aluminum. This two-stage pattern, with a clear interval between peaks, is consistent with the polymorphic phase transformation mechanism reported in the literature.^5,32,33^

The Al@PPh_3_/CuO system exhibits a fundamentally different peak structure. The thermite reaction onset is delayed to ∼543 °C, approximately 50 °C higher than in the native oxide system, because the thin in situ oxide has not yet reached the critical thickness for polymorphic transformation. This delayed onset places the reaction close to the melting point, so the two events overlap significantly. Part of the reaction heat is consumed by aluminum fusion rather than raising the local temperature, which in turn limits CuO decomposition and the oxygen supply available to sustain the reaction.^12^ This accounts for the broader and lower first exothermic peak compared to the native oxide system. Despite the suppressed first-peak, the reaction continues through melting as the exotherm resumes immediately above the aluminum melting point, producing the connected second peak. The two peaks form a nearly continuous exothermic envelope, indicating that the reaction does not fully stall. This contrasts with the native oxide case, where the thick pre-existing shell insulates the molten aluminum until high-temperature diffusion pathways reopen.

The sharp mass drop near 690 °C, observed only in the Al@PPh_3_/CuO samples, is likely due to rapid gas generation as the delayed thermite reaction proceeds vigorously in the presence of molten aluminum. The shift of this mass drop to lower temperature in the richer mixtures is consistent with a greater accumulation of unreacted aluminum during the delay phase, leading to a more violent reaction upon shell failure. The absence of a high-temperature exothermic peak in the Al@PPh_3_/CuO samples suggests that the thermite reaction essentially completes within the 550–700 °C window.

### 3.4 High-Speed Visualization and Burn Rate

Representative high-speed image sequences of the strand burner tests are shown in Figure 11 for 30% Al@PPh_3_ replacement. At 30% replacement, the luminous front propagates continuously through the capillary, consistent with robust self-sustained combustion. Similar self-sustained propagations throughout the tubes were achieved for nanothermites with Al@PPh_3_ replacement up to 50 wt %. The reaction front intensity for the 30 wt % Al@PPh_3_ replacement is comparable to that of 0 wt % (native oxide-passivated Al) in Figure 12(a), although the light intensity is considerably dimmer in the burned zone. At 50 wt % replacement, by contrast, the flame front becomes much less stable, and the luminous zone is more intermittent (see Figure 12(b)), indicating a system close to the propagation limit. Self-sustained propagation cannot be achieved for Al@PPh_3_ replacement above 50 wt % with the current setup. It should be noted that the limit is affected by both the packing density of the powder and the size of the tube.^10^

**Figure 11:**
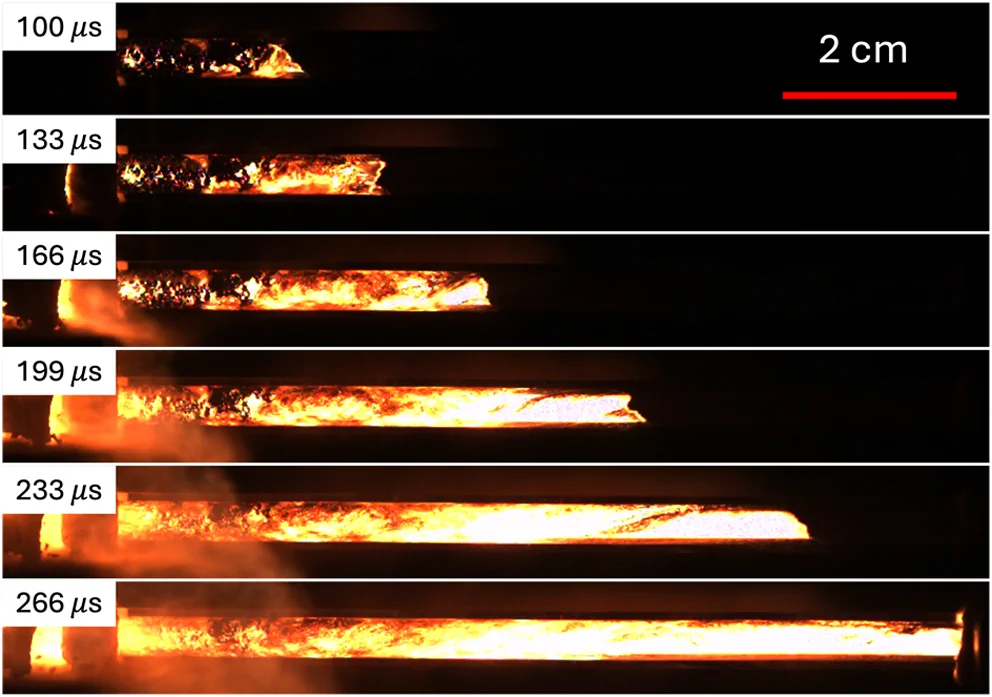
High-speed image sequence of strand burner test at 30 wt % Al@PPh_3_ replacement.

**Figure 12:**
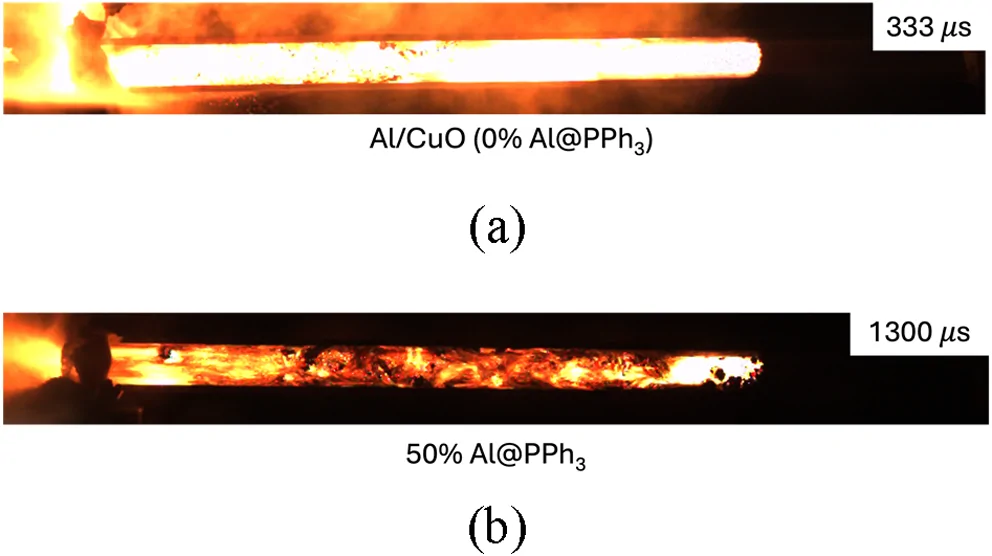
Reaction front structures for (a) Al/CuO nanothermite without Al@PPh_3_, and (b) with 50 wt % Al@PPh_3_ replacement.

Figure 13 presents the burn rates of Al@PPh_3_/CuO nanothermites as a function of Al@PPh_3_ content. The values shown are averaged propagation velocities at near steady-state, and the error bar represents one standard deviation. The 0 wt % condition, corresponding to pure commercial Al/CuO nanothermite, provides a baseline burn rate of about 330 m/s. Replacing 30 wt % of the commercial aluminum with Al@PPh_3_ increases the burn rate to 387 m/s. Further increasing the Al@PPh_3_ fraction causes a pronounced decline, with the burn rate dropping to about 95 m/s at 50 wt % replacement. The dependence is therefore strongly nonmonotonic.

**Figure 13:**
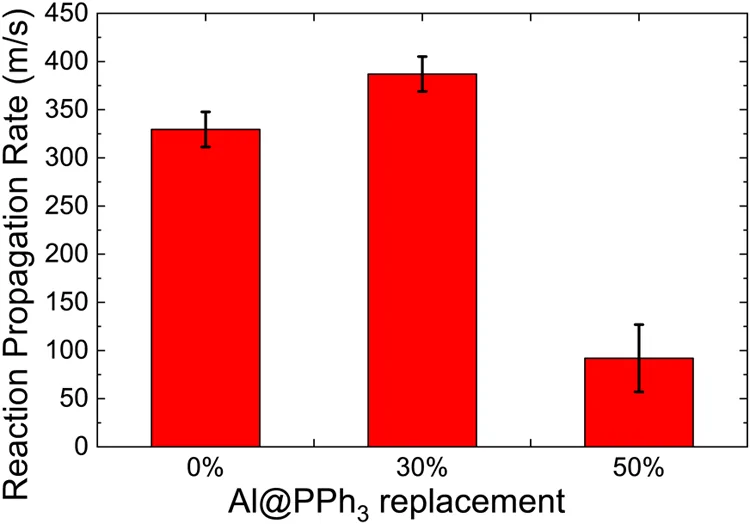
Burn rate of Al@PPh_3_/CuO nanothermites as a function of Al@PPh_3_ replacement fraction at ϕ_Al_ = 1.6.

The improvement at 30 wt % replacement indicates that Al@PPh_3_ can act as a promoter at modest loading. Compared with native alumina, the PPh_3_-derived shell is largely removed at lower temperatures before the thermite reaction initiates. In addition, PPh_3_ volatilization and decomposition during preheating generate gas that may enhance convective transport in the packed bed. This contribution, although it was not measured directly, is consistent with the enhanced burn rate observed at moderate replacement. However, the endothermic PPh_3_ decomposition step absorbs heat and delays aluminum availability simultaneously, so at high Al@PPh_3_ loadings the penalty in the rate of energy delivery outweighs the earlier gas generation. In situ pressure or evolved gas diagnostics should be performed in the future to resolve these individual contributions and to directly compare storage stability with that of conventional oxide-passivated aluminum.

## 4 Conclusions

Triphenylphosphine-passivated aluminum nanoparticles (Al@PPh_3_) were synthesized by a one-pot wet chemical reduction of AlCl_3_ with LiAlH_4_ in refluxing mesitylene at a 1:1 PPh_3_/AlCl_3_ molar ratio, an order of magnitude below previously reported ratios. The product is a crystalline fcc aluminum core with an effective diameter of ∼65 nm, carrying a conformal amorphous shell about 4.6 nm thick. No crystalline Al_2_O_3_ was detected by XRD and no oxide interlayer was resolved by HR-TEM. Solid-state ^31^P NMR shows that intact molecular PPh_3_ does not survive the synthesis, so the shell is a PPh_3_-derived, partly oxidized organophosphorus layer instead of coordinated PPh_3_.

Simultaneous thermal analyses reveal that the reaction characteristics of Al@PPh_3_ differ from those of native oxide-passivated aluminum nanoparticles. In air, the shell is volatilized and decomposed up to ∼450 °C, after which the exposed core oxidizes. Under argon, Al@PPh_3_/CuO mixtures react with an onset near 543 °C, about 50 °C above the native oxide reference. The delayed onset places the reaction close to the aluminum melting point, so the solid-state and molten state exotherms merge into a nearly continuous envelope between 550 and 700 °C, in contrast to the two separated peaks of the native oxide system. The fuel rich case, ϕ_Al_ = 1.6, gave the largest heat release.

In strand burner tests, these Al@PPh_3_ particles were evaluated as a partial replacement for conventional oxide-passivated aluminum in Al/CuO nanothermites. The consequence for propagation is nonmonotonic. At ϕ_Al_ = 1.6, replacing 30 wt % of the commercial aluminum raised the burn rate from ∼330 to 387 m/s, whereas 50 wt % replacement reduced it to ∼95 m/s and self-sustained propagation could not be established above 50 wt %. Aluminum nanoparticles encapsulated in a PPh_3_-derived shell can therefore either promote or retard reaction propagation in an Al/CuO nanothermite, depending on the fraction added. The upper limit of the useful loading range is set by the organic content carried by each particle, so an appropriate shell thickness, thin enough to limit the inert load yet sufficient to passivate the core, is an important design consideration.

Several questions remain open. The bonding geometry at the aluminum/shell interface is unresolved, and the storage stability of Al@PPh_3_ relative to conventional oxide-passivated aluminum remains to be established. Varying the PPh_3_/AlCl_3_ ratio offers a route to tune the shell thickness, which sets both the inert mass and the gas generating material carried by each particle. Even so, this work shows that Al@PPh_3_ can be used in energetic applications. At the right loading, it may even outperform the conventional oxide-passivated aluminum it replaces.
